# Characteristics of Exosomes and the Vascular Landscape Regulate Exosome Sequestration by Peripheral Tissues and Brain

**DOI:** 10.3390/ijms232012513

**Published:** 2022-10-19

**Authors:** William A. Banks, Priyanka Sharma, Kim M. Hansen, Nils Ludwig, Theresa L. Whiteside

**Affiliations:** 1 Geriatric Research Education and Clinical Center, Veterans Affairs Puget Sound Health Care System, Seattle, WA 98108, USA; 2 Division of Gerontology and Geriatric Medicine, Department of Medicine, University of Washington School of Medicine, Seattle, WA 98104, USA; 3 Department of Pathology, University of Pittsburgh School of Medicine, Pittsburgh, PA 15261, USA; 4 University of Pittsburgh Hillman Cancer Center, University of Pittsburgh School of Medicine, Pittsburgh, PA 15232, USA; 5 Amity Institute of Virology and Immunology, Amity University, Noida 201313, Uttar Pradesh, India; 6 Department of Oral and Maxillofacial Surgery, University Hospital Regensburg, 93053 Regensburg, Germany; 7 Departments of Immunology and Otolaryngology, University of Pittsburgh School of Medicine, Pittsburgh, PA 15260, USA

**Keywords:** exosomes, liver, kidney, brain, spleen, lung, lipopolysaccharide, inflammation, wheatgerm agglutinin, mannose-6 phosphate receptor

## Abstract

Exosomes mediate intercellular communication, shuttling messages between cells and tissues. We explored whether exosome tissue sequestration is determined by the exosomes or the tissues using ten radiolabeled exosomes from human or murine, cancerous or noncancerous cell lines. We measured sequestration of these exosomes by the liver, kidney, spleen, and lung after intravenous injection into male CD-1 mice. Except for kidney sequestration of three exosomes, all exosomes were incorporated by all tissues, but sequestration levels varied greatly among exosomes and tissues. Species of origin (mouse vs. human) or source (cancerous vs. noncancerous cells) did not influence tissue sequestration. Sequestration of J774A.1 exosomes by liver involved the mannose-6 phosphate (M6P) receptor. Wheatgerm agglutinin (WGA) or lipopolysaccharide (LPS) treatments enhanced sequestration of exosomes by brain and lung but inhibited sequestration by liver and spleen. Response to LPS was not predictive of response to WGA. Path and heat map analyses included our published results for brain and found distinct clusters among the exosomes and the tissues. In conclusion, we found no evidence for a universal binding site controlling exosome-tissue interactions. Instead, sequestration of exosomes by tissues is differentially regulated by both exosomes and tissues and may be stimulated or inhibited by WGA and inflammation.

## 1. Introduction

Exosomes, a subset of small extracellular vesicles (EVs), ranging in diameter from 30–150 nm, are produced by normal and malignant cells and are found in all body fluids [1,2,3]. Exosomes are formed as inverse membrane invaginations inside multivesicular bodies (MVBs) and thus originate from the endosomal cell compartment [4]. The topography of exosome surface proteins and their molecular content resemble that of the parent cells [3] which, in part, explains why they can serve as surrogates of the producer cell.

Exosomes circulate freely and are taken up by a variety of tissue cells, resulting in the phenotypic and functional re-programming of recipient cells [4,5]. They can convey peptides, proteins, and genetic materials between cells without a loss of function and thus qualify as a highly effective intercellular communication system [4,5,6,7]. In health and disease, exosomes exert profound and varied biological effects on a broad spectrum of cells in the nearby and distantly located tissues [8,9]. For example, exosomes convey signals to immune cells that a tissue injury has occurred, stimulating trafficking and entry of immune cells into tissues [10]. In cancer, they can prepare the tissue’s endothelial bed for metastasis [11], and because of their propensity for cell reprogramming, exosomes are emerging as disease biomarkers in cancer, infections, or autoimmune dysfunctions [12,13,14]. The mechanisms underpinning these various activities of exosomes remain obscure.

Currently, only a limited understanding exists of exosome trafficking, although general mechanisms of exosome retention by tissues have been described [15]. Vesicular internalization follows the exosome attachment to binding sites on the cell membrane of the target tissue. The binding sites on exosomes and target recipient cells are mainly glycoproteins and glycolipids, with integrins located on exosomes playing a particularly important role as binding sites [16]. To enter into the brain, exosomes first bind to the brain endothelial cells comprising the blood–brain barrier (BBB), inducing vesicularization (transcytosis) [17].

A large number of questions about exosome-tissue interactions remain unanswered, including: (i) the potential for preferential or selective sequestration of exosome subsets by various target cells and tissues; (ii) the role of specific cellular receptors in exosome sequestration; (iii) the previously described ready acceptance of human and murine exosomes injected into mice [18]; (iv) differences in the incorporation rate of tumor-derived exosomes vs. non-malignant exosomes; (v) the sequestration rates of exosomes produced by immune cells relative to exosomes derived from non-immune cells. In addition to exosome origins and associated attributes, exosome-tissue interactions might be modulated by conditions induced in the tissue microenvironment such as, e.g., local inflammation or overexpression of certain receptor/ligands in targeted tissues. To answer these questions, we examined the in vivo sequestration by murine kidney, lung, liver, and spleen of radioactively labeled human and mouse exosomes carefully selected as products of various malignant or non-malignant cells. We compared the sequestration data with results previously reported by us for mouse brain, where the same mice were injected with the same ten radiolabeled exosome populations [18]. Our study provides new insights into entry and incorporation of exosomes by various peripheral tissues and brain that pave the way for the future therapeutic applications.

## 2. Results

*Exosome characteristics***:** Human or mouse exosomes were obtained from supernatants of cultured malignant or non-malignant cells [18] (see Table 1). The characterization procedure used for all the vesicles harvested from supernatants of the ten cell lines is illustrated in Figure S1, in this case using vesicles from supernatants of PCI-30 cell line. The protein concentrations of isolated exosomes in the recovered SEC fractions #4 ranged between 60 and 120 µg/mL supernatant. Supernatants of HaCat and NIH-3T3 (noncancerous cell lines) yielded fewer vesicles than supernatants of malignant cells (data not shown). The vesicles we isolated by the same ultrafiltration/SEC method from 10 different cell lines ranged in size from 30–200 nm (mean diameter 103 nm (SD 14.4) and displayed a characteristic vesicular morphology by TEM. All vesicles carried tetraspanins (CD9 CD63, CD81), although CD9 was absent from some exosomes. All exosomes were TSG101+ and ALIX+, suggesting their endocytic origin, and did not carry cytoplasmic proteins, calnexin or Grp94. According to the recently adopted nomenclature, these vesicles are “small extracellular vesicles” [12], here referred to as “exosomes”.

As also previously noted [18] when exosomes produced by three cell lines, HaCaT, MEL526 and PCI-30, and by a primary T cell line were examined for surface expression of CD46, integrin αVβ3, integrin αVβ6, and ICAM 1 by on-bead fluorescence, the RFI values differed for each exosome population (data not shown; see [18]). All four exosome populations carried surface CD46, at different expression levels. Non-malignant cell-derived exosomes (HaCaT and primary T cells) did not carry αVβ3 and had lower levels of αVβ6 than exosomes produced by malignant cells. All exosomes were uniformly rich in in ICAM1.

### 2.1. Sequestration of Radiolabeled Exosomes by Tissue

#### 2.1.1. Liver

**Figure 1:**
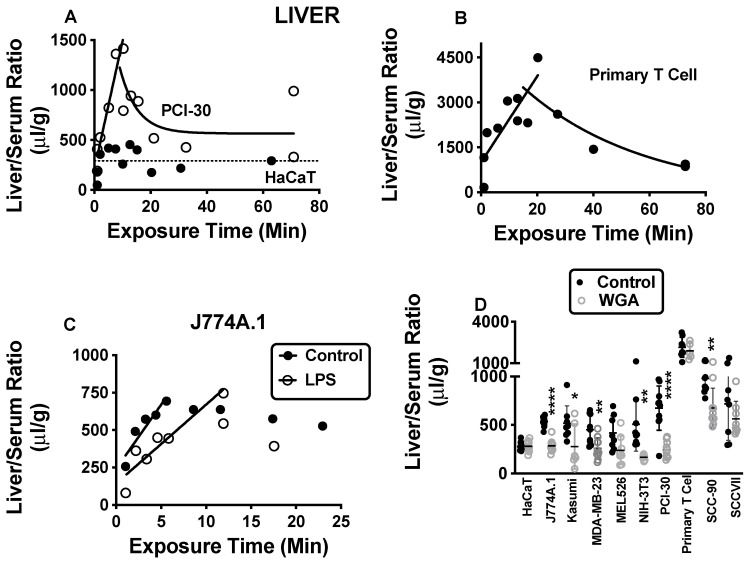
Liver sequestration of exosomes. In (**A**), the pattern as exemplified by PCI-30 for those exosomes that were sequestered by liver in a time-dependent manner (open circles, solid line, n = 13). That panel also illustrates HaCaT as an example of exosomes whose sequestration levels did not vary with time (solid circles, dashed line, n = 11). In (**B**), the most robustly sequestered exosome was derived from primary human T-cells (n = 13). In (**C**), the typical sequestration pattern as exemplified by J774A.1 for exosomes whose liver sequestration was inhibited by LPS treatment (n = 9 for control and for LPS). In (**D**), six exosomes (J774.1: n = 10 for control, n = 10 for WGA; NIH-3T3: n = 9 for control, n = 9 for WGA; Kasumi: n = 8 for control and 6 for WGA; MDA-MB-23: n = 9 for control, n = 10 for WGA; PCI-30: n = 10 for control; n = 10 for WGA; SCC-90: n = 10 for control; n = 10 for WGA) had sequestration that was increased by WGA. See Figure S2 for exosomes not shown here. Regression analysis was used for panels (**A**–**C**) and *t*-tests for panel (**D**). * *p* < 0.05; ** *p* < 0.01; **** *p* << 0.001.

**Figure 2:**
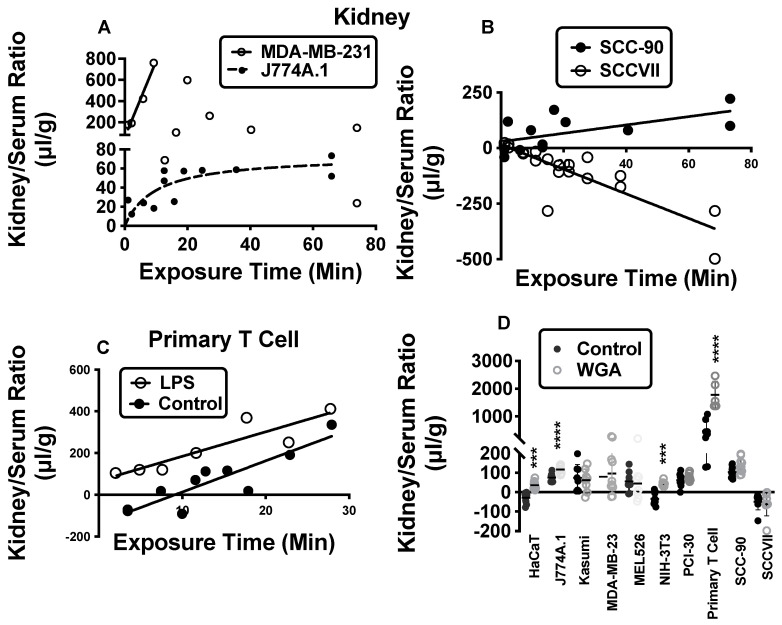
Kidney sequestration of exosomes. In (**A**), the two patterns of exosome sequestration for kidney: a hyperbolic pattern as exemplified by J774A.1 (n = 13) and an increase followed by decrease as exemplified by MDA-MB-231 N = 11). In (**B**), the pattern for SCC-90 (n = 13) and for SCCVII (which had negative values, n = 20) is shown. In (**C**), the increased sequestration of exosomes with LPS treatment for primary human T-cell exosomes (n = 13). In (**D**), four exosomes (HaCaT and J774A.1: n = 10 for all groups; NIH-3T3: n = 9 for control; n = 9 for WGA; Primary T cell: n = 8 for control, n = 5 for WGA) had sequestration that was increased by WGA (mean +/− SE). See Figure S2 for exosomes not shown here. Regression analysis was used for panels (**A**–**C**) and *t*-test in panel (**D**). *** *p* < 0.001; **** *p* << 0.001.

**Figure 3:**
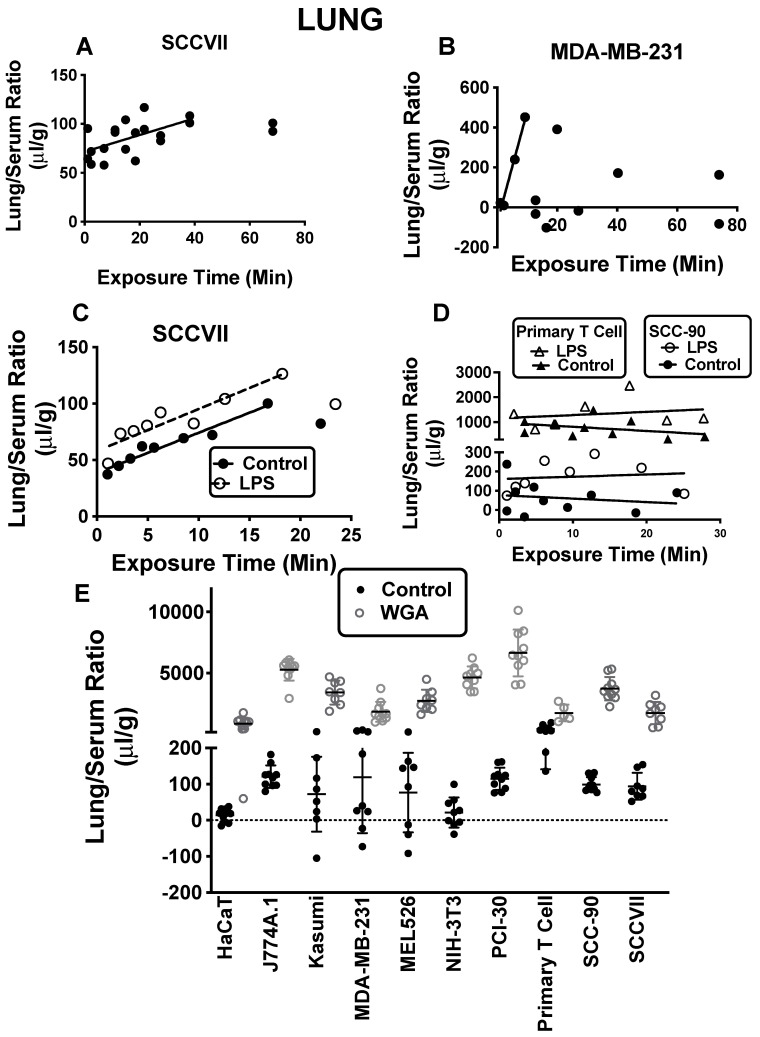
Lung sequestration of exosomes. In lung, only SCCVII and MDA-MB-231 showed time-dependent sequestrations. SCCVII (n = 20) showed a hyperbolic pattern (**A**) and MDA-MB-231 (n = 12) showed a decline following its initial increase (**B**). Modest increases were induced by LPS for exosomes whose sequestration either increased ((**C**); n = 9/group) or was stable ((**D**); n = 10 for each control; n = 7 for Primary T cell LPS; n = 8 for SCC-90) over time. In (**E**), WGA dramatically increased sequestration of each of the exosomes to a statistically significant level (n = 10 for control and WGA for HaCaT, J774A.1, PCI-30, SCC-90, and MDA-MB-231 WGA; n = 9 for control and WGA for MEL526 and NIH-3T3; n = 9 for controls for MDA-MB-231 and Primary T cell; n = 8 for control and WGA for Kasumi and SCCVII; n = 5 for WGA for Primary T cell). See Figure S2 for exosomes not shown here. Regression analysis was used in panels (**A**–**D**) and *t*-tests in panel (**E**). *p* << 0.001 for each comparison.

**Figure 4:**
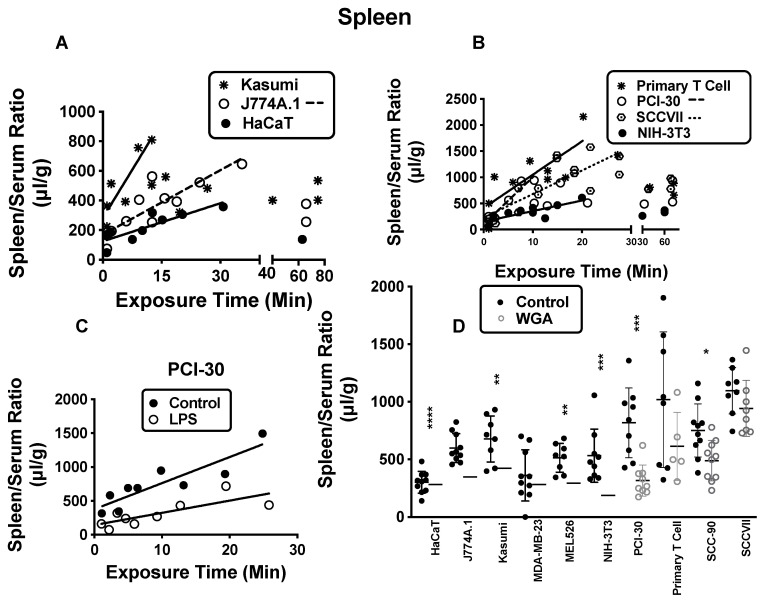
Spleen sequestration of exosomes. In (**A**) (Kasumi, asterisk with solid line n = 13; J774A.1, open circle with dashed line, n = 13; HaCaT, solid circle with solid line, n = 11) and (**B**) (Primary T cell asterisk with solid line, n = 13; PCI-30, open circle with dashed line, n = 13; SCCVII, targeted circle with dotted line, n = 20; NIH-3T3, solid circle with solid line, n = 12), the patterns for spleen for different exosome types are shown. In (**C**), a decrease in sequestration with LPS as exemplified by PCI-30 is shown (n = 9/group). In (**D**), six exosome types (n = 10 for control and WGA for J774A.1 and SCC-90; n = 9 for control and WGA for NIH-3T3 and PCI-30; n = 8 for control and WGA for Kasumi and MEL526) whose sequestrations were suppressed by WGA are shown. * *p* < 0.05; ** *p* < 0.01, *** *p* < 0.001, **** *p* << 0.001. See Figure S2 for exosomes not shown here. Regression analysis was used in panels (**A**–**C**) and *t*-tests in panel (**D**).

Figure 1 shows representative results for sequestration by liver of exosomes produced by human keratinocytes (HaCaT), human tumor cells (PCI-30), mouse macrophages (J774A.1), and primary human T-cells. Figure S2 shows exosomes not illustrated in Figure 1, Figure 2, Figure 3 and Figure 4. For eight exosomes, a sharp increase in the liver/serum ratios peaked at 10–20 min and was followed by a decline, except for exosomes produced by HaCaT and SCC-90, where the ratios were flat over time (Figure 1A). The largest value was for primary human T-cell exosomes (Figure 1B). Sequestration of PCI-30 (Figure 1A), MDA-MB-231, SCC-90 and SCCVII increased to the 1200–1500 μL/g range, whereas sequestration of J774A.1, Kasumi, and NIH-3T3 peaked at 800–900 μL/g; MEL526 peaked at about 500 μL/g. Thus, while 8/10 exosomes showed similar patterns for liver sequestration, the degree of sequestration varied almost 10 fold.

LPS had no statistically significant effect on liver sequestration of exosomes from HaCaT, Kasumi, MDA-MB-231, primary human T-cell, or SCC-90. LPS treatment decreased liver sequestration of exosomes from J774A.1 (Figure 1C), MEL526, NIH-3T3, and SCCVII, and there was a trend for a decrease for exosomes from PCI-30. For NIH-3T3 and SCCVII, the influx rate was significantly decreased, whereas for MEL526, the intercept was shifted downward. J774A.1 showed a hybrid pattern with the intercept shifted downward and the slope decreased, although there was a statistical effect only for the decrease in intercept (Figure 1C). WGA caused a significant decrease in sequestration of exosomes from J774A.1, Kasumi, MDA-MB-23, NIH-3T3, PCI-30, and SCC-90 by the liver (Figure 1D). The only effect M6P had throughout the entire study was a slight increase in uptake of J774A.1 by liver (537 +/−16.9, n = 10 vs. 619 +/−28, n = 8, t = 2.64, df = 16, *p* = 0.018).

#### 2.1.2. Kidney

For exosome sequestration by kidney (Figure 2), MDA-MB-231 showed a rapid increase and subsequent decline, while a hyperbolic relation was seen for J774A.1 (Figure 2A). Sequestration of SCC-90 exosomes (Figure 2B) and primary human T-cell exosomes (Figure 2C) was linear, whereas SCCVII showed an inverse relation with exposure time and negative kidney/serum ratios (Figure 2B). The negative values for SCCVII indicate that it is sequestered less well by the kidney than serum albumin. There was no correlation between kidney/serum ratios and exposure time for HaCaT, Kasumi, MEL526, NIH-3T3, or PCI-30 exosomes, although the ratios were 20–100 μL/g higher than the ratios for albumin (see Figure S2).

LPS had an effect on kidney sequestration for only 3 exosomes derived from primary human T-cell (Figure 2C), SCC-90, and SCCVII. For SCCVII, kidney/serum ratios remained negative in LPS-treated mice, but at values closer to zero than in the controls. WGA increased sequestration of exosomes from HaCaT, J774A.1, NIH-3T3, and primary human T-cells (Figure 2D). M6P had no effect on any of the 10 exosomes.

#### 2.1.3. Lung

Figure 3 illustrates sequestration of various exosomes by lung. Only SCCVII and MDA-MB-231 accumulated in lung as a function of time (Figure 3A,B). Other exosomes had low lung/serum ratios of 10–100, except for primary human T-cell exosomes, which had ratios of about 500 μL/g (Figure S2).

LPS increased lung sequestration of exosomes to a modest, but statistically significant degree for Kasumi, MDA-MB-231, primary human T-cell, SCC-90 and SCCVII exosomes, but not for the other exosomes (see examples in Figure 3C,D). LPS decreased exosome sequestration for J774A.1 (data not shown). M6P had no effect on lung sequestration of exosomes, but WGA dramatically increased sequestration for all exosomes (Figure 3E).

#### 2.1.4. Spleen

Seven of the exosomes accumulated as a function of time by the spleen, the exceptions being MEL526, MDA-MB-231, and SCC-90, which nevertheless had spleen/serum ratios averaging about 350–600 μL/g above those for radioactive albumin (Figure S2). Figure 4A,B show the relationships between the spleen/serum ratios and time for all of the exosomes that were sequestered over time except MDA-MB-231, which closely resembled the pattern for Kasumi. In general, a plateau or a fall followed after about 20–30 min.

LPS caused a significant decrease in the sequestration by spleen for exosomes produced by J774A.1, MDA-MB-231, MEL526, NIH-3T3, PCI-30, primary human T-cells, and SCCVII, but not for HaCaT, Kasumi, or SCC-90 exosomes (see Figure 4C for representative data with PCI-30 exosomes). M6P had no effect on sequestration of any exosomes, whereas WGA induced a statistically significant decrease for 6 exosomes (Figure 4D).

### 2.2. Single Time-Point Analysis of Sequestration

**Figure 5:**
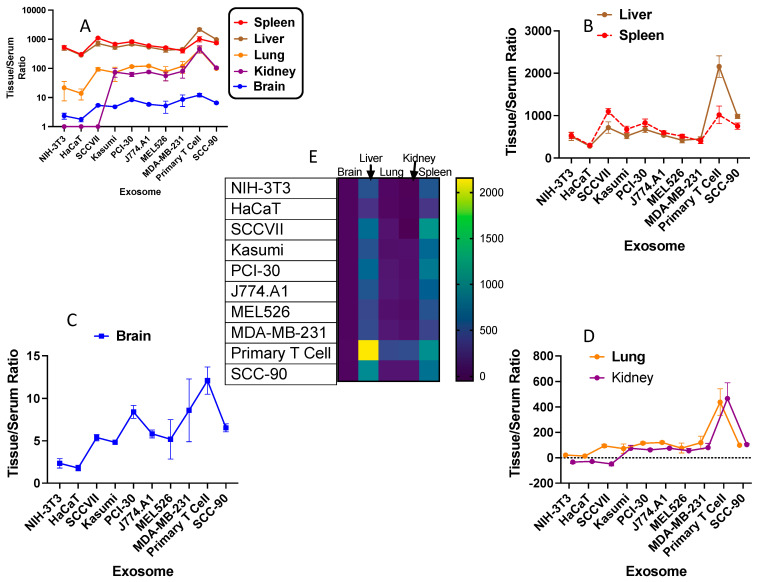
Comparison of exosome sequestration by various tissues. In (**A**)**,** the tissue/serum ratios for all tissues and all exosomes are shown in a logarithmic scale. NIH3T3, HaCAT, and SCCVII for kidney all had negative values but are plotted as a value of one. In (**B**–**D**), exosome sequestration by the five tissues is plotted in an arithmetic scale. In (**E**)**,** the heat map for all the data is presented. The data are means with SD and were obtained from mice used as controls for the WGA experiments which were studied 20 min after iv injection of the labeled exosome. Units in all panels are μL/g of tissue.

We next assessed whether there were similarities of uptake among the tissues for exosomes (Figure 5 and Table 2). For this assessment, we used the control data from the experiment that assessed the effect of WGA on uptake. Figure 5A shows all tissues and exosomes with tissue/serum values plotted against a logarithmic axis. The kidney values for NIH-3T3, HaCaT, and SCCVII were negative, but appear in Figure 5A as values = 1. Spleen and liver show the highest ratios of sequestration, i.e., have the highest tissue/serum values, for all exosomes. Lung and kidney have a moderate ratio, while brain has the lowest ratio of for all exosomes. Figure 5B–D show the tissue/serum ratios for each of five tissues for sequestration of all exosomes plotted against an arithmetic scale to emphasize differences in tissue sequestration values. Figure 5E is a heat map of sequestration.

### 2.3. Results of Path Analysis

**Figure 6:**
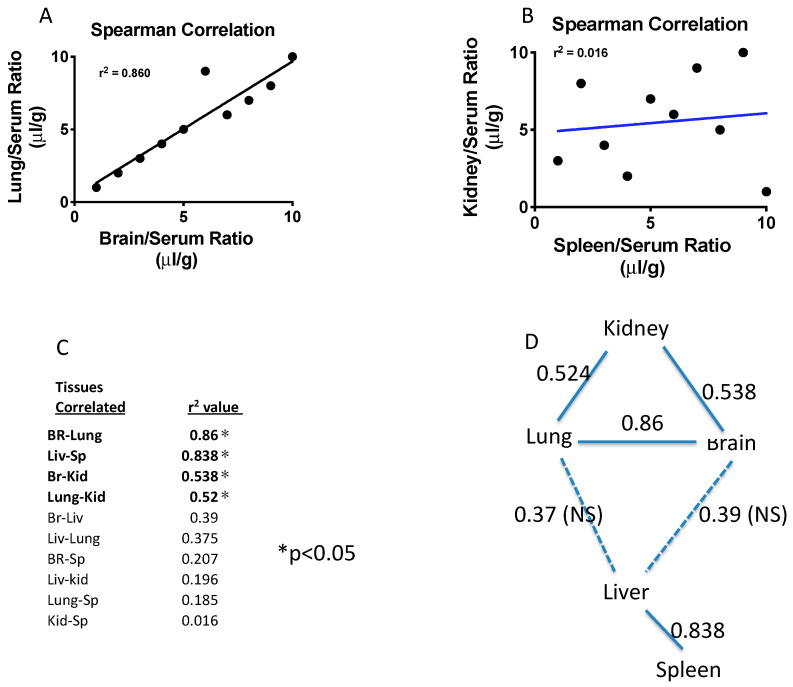
Path analysis for tissues. In (**A**,**B**), examples of correlations of the ten exosomes among paired tissues. In (**C**), a list of all r^2^ values for various combinations of tissues. * indicates those paired tissues that showed a statistically significant correlation. In (**D**), the path analysis for the five tissues studied. Liver-spleen form one strongly related cluster, whereas lung-brain show a second strongly related cluster, with kidney also correlated to lung and brain.

The observed differences in exosome sequestration ratios between tissues were further evaluated by path analysis. The analysis was performed for the four tissues as well as the previously published data for brain, which were from the same animals [18] The first step in path analysis was to determine the correlations among all tissue pairs using Spearman correlations. Figure 6A shows the pair with the strongest Spearman correlations (lung/serum ratios vs. brain/serum ratios) and Figure 6B the weakest Spearman correlation (spleen/serum ratios vs. kidney/serum ratios). Figure 6C shows the tabulated r^2^ values for each of the possible tissue comparisons with those having a statistically significant correlation indicated by an “*”. Figure 6D is a graphic presentation of the path analysis showing the r^2^ values, where solid lines represent r^2^ values that were statistically significant and dashed lines are those that were not significant. A higher r^2^ value results from a greater statistical correlation between the pair of tissues (that is, how well sequestration by one tissue can predict the degree to which it will be sequestered by another). The results show two significant clusters of relationships. Brain and lung are highly correlated, and lung and brain are also correlated with kidney, although less so. Liver and spleen form another highly correlated cluster. The least related pair is that of kidney and spleen with an r^2^ = 0.016.

**Figure 7:**
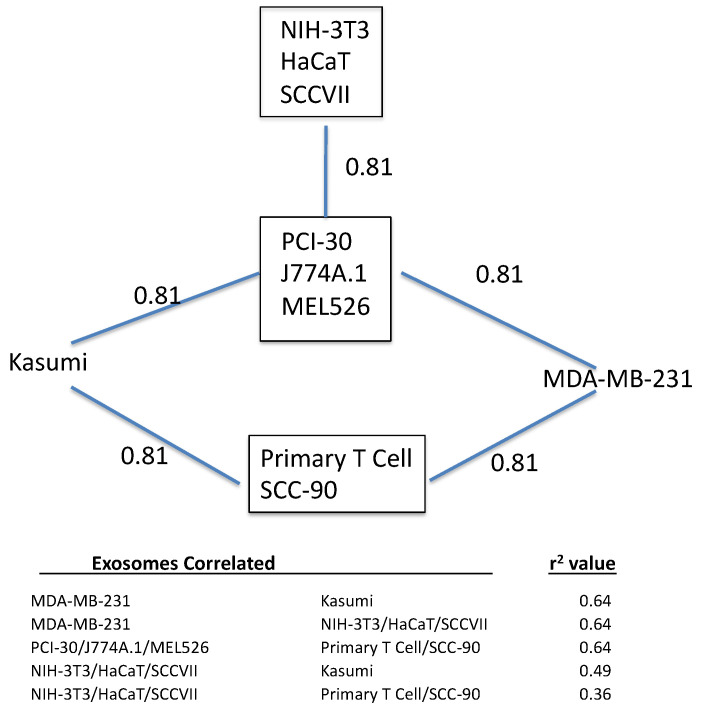
Path analysis for exosomes. Exosomes listed in boxes correlated perfectly (r^2^ = 1.0) with other graphed relations found to be statistically significant. Values below the figure are remaining possible correlations and their r^2^ values, none of which were significant.

Path analysis based on exosome pairs is shown in Figure 7. For exosome pairs, there were only 5 tissue/serum ratios (one for each of the five tissues) for constructing the regression lines, giving only 3 degrees of freedom. In contrast, path analysis for the tissue had 10 tissue/serum ratios (one for each exosome) and so 8 degrees of freedom. Therefore, path analysis for exosome clustering is much less detailed. Three sets of exosomes were perfectly correlated (r^2^ = 1.0): (a) NIH-3T3, HaCaT, and SCCVII; (b) PCI-30, J774A.1, and MEL526; (c) Primary T cell and SCC-90. Statistically significant relations with Kasumi and MDA-MB-231 are also shown. The table in Figure 7 shows the r^2^ values among the exosome pairs that were not statistically significant.

**Figure 8:**
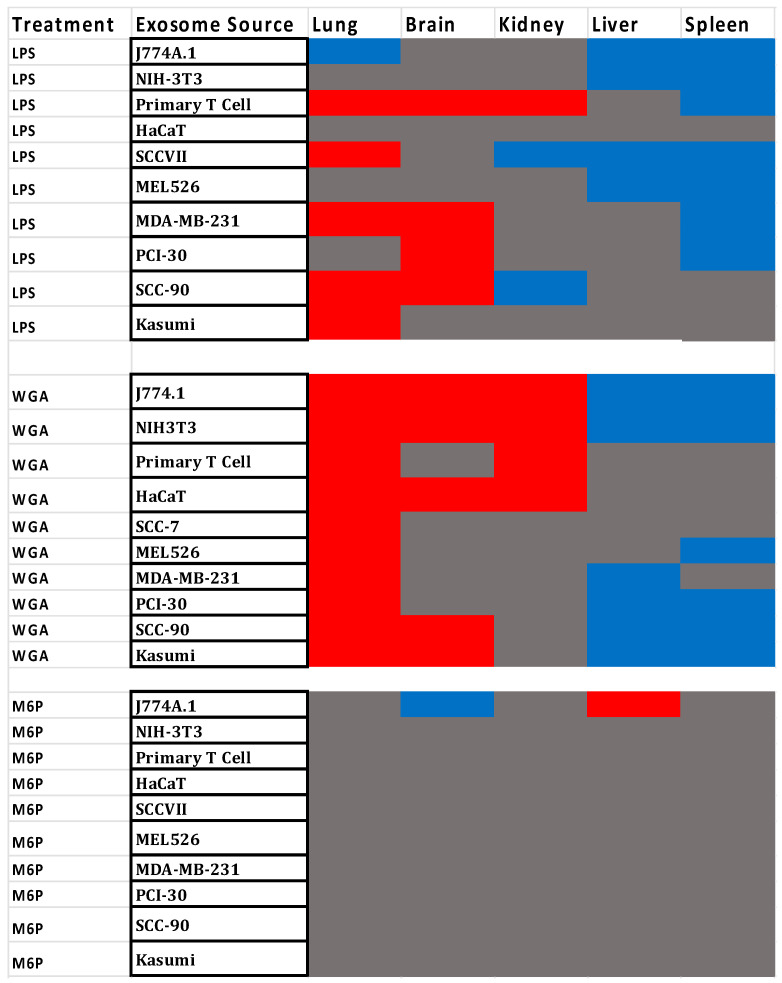
Heat map correlating changes in exosome sequestration following LPS, WGA or M6P treatments. Blue color indicates that LPS, WGA, or M6P induced a statistically significant decrease in sequestration of the exosomes, and red color indicates that LPS, WGA, or M6P induced a significant increase in sequestration of the exosomes. Grey indicates that LPS, WGA, or M6P did not produce a statistically significant alteration in the exosome sequestration. Liver-spleen show strong similarities to one another, whereas lung, brain, and kidney form another group.

The heat map in Figure 8 shows the effects on LPS, WGA, and M6P treatments on the sequestration of exosomes by various tissues, with gray indicating no effect of treatment on exosome sequestration; blue indicating that treatment decreased the exosome tissue sequestration; and red indicating that treatment increased exosome tissue sequestration. The data obtained for brain and peripheral tissues are derived from the same mice, although the data for brain have been previously published [18]. The heat map shows two main clusters, with liver and spleen responding similarly to LPS or WGA treatment by decreased exosome sequestration, kidney and lung responding to LPS or WGA treatment by increased exosome sequestration, and brain responses being more similar to those of kidney and lung.

## 3. Discussion

Trafficking of EVs in the circulation and their entry into the brain and peripheral tissues is of key significance for intercellular communication these vesicles mediate in health and disease (6). Despite physiological and pathological importance of EVs, only limited information exists regarding their pharmacokinetics and sequestration by different tissues after systemic administration [19,20,21]. Here, we examined the ability of four peripheral murine tissues: lung, kidney, liver, and spleen to sequester ten different types of exosomes produced by human and mouse cancerous or noncancerous cells. We examined sequestration when the innate immune system was stimulated with LPS and also after WGA treatment. M6P was used to block exosome sequestration via the M6P receptor. We compared these results with the previously published data for sequestration by brain of the same ten exosome types injected in the same mice [18].

We reasoned that exosome sequestration by any tissue is dependent on an interaction between the glycoproteins/glycolipids on the exosome surface and their counterparts expressed on cell surfaces of the target tissue. The abundance of the available binding sites on the exosome surface is expected to control the rate of exosome sequestration by tissues. Similarly, the abundance of these binding sites in the tissue or its vascular bed should control the rate of exosome sequestration by this tissue. Spleen and liver showed the most abundant sequestration of exosomes, and in 7/10 cases brain sequestered the least amount of exosomes (Figure 5). This observation is consistent with the barrier functions of the brain’s vascular bed [22]. The three cases (NIH-3T3, HaCaT, SCCVII) where values for kidney sequestration were negative (that is, retention of albumin was greater than retention of exosomes) we suggest is explained by an absence or low level of binding sites for those exosomes in the kidney. Thus, sequestration would depend on leakage and, since exosomes are about 10 times the diameter of an albumin molecule, the rate of leakage for exosomes would be less than that for albumin. The primary T cell exosomes usually showed the greatest tissue sequestration, and HaCaT exosomes tended to show the lowest.

A comparison of various tissues for the sequestration of exosomes in the presence/absence of LPS, WGA and M6P provides additional key information about exosome-tissue interactions. For example, if exosome sequestration relied on a single, universal binding site that determined sequestration, then all exosomes would be sequestered by all cells in proportion to the expression of that binding site. To the extent that the expression of various binding sites differs among exosomes, target tissues, or both, a corresponding variety among the sequestration patterns would occur. WGA affects binding to glycoproteins expressing sialic acid and N-acetyl-D-glucosamine and M6P binds to the mannose-6-phosphate receptor [23,24,25,26]. Thus, an effect of WGA or M6P indicates the involvement in sequestration of those respective binding sites. LPS stimulates the innate immune system, which alters many cell membrane receptors and transporters, including expression of the selectins [27,28]. Thus, an effect of LPS indicates a binding site in the target tissue responsive to the innate immune system. Whereas WGA and M6P were co-injected with the exosome and so could be acting on binding sites at either the tissue or the exosome, mice were pretreated with LPS and so its site of action is much more likely to be at the tissue than the exosome.

With the exception of kidney retention of SCCVII, NIH-3T3, and HaCaT, all exosomes under all conditions entered each tissue to an extent exceeding that of albumin, indicating *tissue sequestration*. However, the patterns of sequestration of exosomes were characterized by variety rather than uniformity. Sequestration ranged from a high of 4500 μg/mL for primary human T-cell exosomes by liver to nondetectable levels for SCCVII exosomes in kidney. There was no discernable effect of species (i.e., mouse vs. human) or source (i.e., cancerous vs. noncancerous cell line type) on the degree of sequestration rate, sequestration patterns, or responses to LPS, WGA, or M6P. For example, WGA decreased sequestration of some exosomes, but increased sequestration of other exosomes. The response of an exosome to WGA did not indicate whether it would also respond to LPS. These findings show *there is no universal receptor or binding site that controls exosome sequestration.*

Our results favor a view that binding sites vary among exosomes and among tissues, producing novel patterns of exosome/tissue interactions. In liver, for example, WGA had an effect on six exosomes and LPS had an effect on four, but only two exosomes were affected by both WGA and LPS. This illustrates the occurrence of 4 categories of binding sites: those affected by the innate immune system and involving sialic acid or N-acetyl-D-glucosamine, those not affected by the innate immune system but involving sialic acid and N-acetyl-D-glucosamine, those affected by the innate immune system but not involving sialic acid or N-acetyl-D-glucosamine, and those not affected by the innate immune system nor involving sialic acid or N-acetyl-D-glucosamine.

There were, however, some subtle patterns to exosome sequestration. Exosomes from human primary T cells had high sequestration in all tissues. Exosomes clustered into the lung-brain and the liver-spleen groups, as noted above. In contrast to exosomes, the tissues did show some consistency in that liver and spleen responding to LPS or WGA had decreased exosome sequestrations, whereas lung and brain had increased sequestrations. For most exosomes, liver had the greatest sequestration that peaked 10–20 min after injection and then declined. This is similar to the findings reported by Charoenviriyakul et al. [21]. The high sequestration of exosomes suggests the liver is the major clearance site for exosomes, and a subsequent decline in sequestration indicates that the liver releases exosomes after their sequestration, although it is unclear if those were intact exosomes, exosomes modified by the liver, or degradation products. 

As already noted, both path analysis (Figure 6) and heat map analysis (Figure 8) showed clustering of lung and brain responses and another clustering of liver and spleen. Path analysis indicated that the ranking of exosomes from low to high correlated well between lung and brain and between liver and spleen. As an example, if an exosome was robustly sequestered by brain, its sequestration by lung would also likely be robust. Heat map analysis showed that WGA or LPS treatments tended to increase exosome sequestration by lung and brain, but decreased exosome sequestration by liver and spleen. The strength of this correlation is highlighted by path analysis, which is based on baseline sequestration rates, whereas the heat map in Figure 8 is based on responses to WGA, LPS, and M6P. Thus, we conclude that the tissue-exosome interacting receptor combinations may be similar between lung and brain and between liver and spleen.

Why lung-brain would have opposite directional responses than liver-spleen to LPS and WGA is unclear. LPS could have a differential effect on expression of receptors key to exosome sequestration. By analogy, LPS treatment increases immune cell trafficking into brain, but decreases immune cell sequestration by spleen [29]. In the current experiments, mice but not exosomes were treated with LPS prior to injection, and so the LPS effects here are mediated at the tissue level. WGA affects sequestration through the process of adsorptive endocytosis, which has the hallmark of increasing rather than inhibiting sequestration of its ligands through stochiastic binding mechanisms [30].

The only exosome-tissue combination affected by M6P was J774A.1 and liver; and in the previous study of exosome sequestration by brain, only J774A.1 was affected by M6P. This suggests that a mannose-6 phosphate receptor is important in the sequestration of J774A.1 exosomes by both brain and liver. Future analysis will likely show other receptors shared by lung and brain as well as liver and spleen. Similarly, path analysis showed exosome clustering (Figure 7). Because statistical analysis was less robust for exosome clustering than for tissue clustering, results are less fine-grained. Nevertheless, the results indicate that groups of exosomes are likely to have similar binding sites. This is consistent with the work showing that distinct integrin expression patterns differentiated exosomes metastasizing to lung from those metastasizing to liver [11].

In comparison to liver, kidney had low levels of sequestration, suggesting it is not a major site of clearance for circulating exosomes. Kidney showed the greatest number of sequestration patterns, including a peak and decline (like liver), linear, flat or hyperbolic, and the only exosome with a retention lower than that of albumin (SCCVII). The three exosomes for which kidney retention was less than that of albumin (NIH-3T3, HaCaT, SCCVII) were also shown by path analysis in Figure 7 to cluster. Kidney had the fewest exosomes whose sequestration was altered by LPS (n = 3) or WGA (n = 4). Lung also had low sequestration and most exosomes had sequestration patterns that were flat, indicating that the exosomes had rapidly reached equilibrium. Lung showed the greatest difference in response to WGA vs. LPS, as all exosomes responded dramatically to WGA increasing their degree of sequestration by 30–50 fold. Spleen had levels of sequestration intermediate between the high of liver and the low of kidney and lung. Sequestration of the immune cell derived exosomes (J774A.1, human primary T cells, and Kasumi) was not favored by the spleen.

## 4. Materials and Methods

*Exosome isolation:* Human or mouse exosomes were obtained from supernatants of cultured malignant or non-malignant cells [18] (Table 1). All cell lines were cultured in media supplemented with fetal bovine sera that were depleted of exosomes by ultracentrifugation at 100,000× *g* for 3 h. All exosomes were isolated from concentrated supernatants, which were pre-cleared by the low speed centrifugation, ultrafiltered using 0.22 µm filters and separated by size exclusion chromatography (SEC) as previously described [12,31]. Vesicles collected in fraction #4 were characterized for size and concentration by nanoparticle tracking analysis (NTA) using NanoSight 300 (Malvern, UK). The vesicles were diluted in ddH_2_O and then the video image was captured at the camera level of 14. The captured videos were analyzed using NTA software, maintaining the screen gain and the detection threshold at 1 and 5, respectively. To determine mean particle size/concentration in each sample, five measurements were obtained and averaged. Vesicular morphology and diameter of vesicles were measured by transmission electron microscopy (TEM) performed at the Center for Biologic Imaging at the University of Pittsburgh as previously described [12]. Briefly, freshly prepared vesicles were placed on a copper grid coated with 0.125% formvar in chloroform. The vesicles on copper grids were stained with 1% (*v*/*v*) uranyl acetate in ddH2O and visualized using TEM (model JEOL JEM-1011). The presence of endocytic markers (TSG101, ALIX), the absence of cytoplasmic proteins such as Grp94 or calnexin, and the enrichment in tetraspanins (CD9, CD63, CD81) were determined by Western blots (WBs) as decribed [12]. The primary antibodies used in WBs were specific for: TSG101 (1:500, #PA5-31260), CD9 (1:500, #10626D) from Thermo Fisher; ApoB (1:2000, #20578-I-AP) from Protein Tech; ALIX (1:500, #2171S), Calnexin (1:1000, #2433) or Grp94 (1:1000, #2104T) from Cell Signaling Technology. The HRP-conjugated secondary antibody (1:5000, Pierce, Thermo Fisher) was used for 1 h at room temperature (RT). The blots were developed using ECL detection reagents (GE Healthcare Biosciences). In some cases, when sufficient vesicle numbers were available, their functions were also evaluated, such as the ability of tumor-derived exosomes to induce apoptosis in activated human T cells [12].

*Culture of primary human T cells:* Peripheral blood was obtained from healthy volunteers who signed a consent form approved by the Institutional Review Board (IRB no. 0403105). Venous blood was collected in heparin tubes and was processed immediately for recovery of peripheral blood mononuclear cells (PBMC) by centrifugation on Ficoll-Paque Plus gradients (GE Healthcare Bioscience, Pittsburgh, PA, USA). The recovered cells were washed in RPMI medium (Lonza, Basel, CH) supplemented with 10% (*v*/*v*) exosome depleted FBS (Gibco, Thermo Scientific, Pittsburgh, PA, USA) and T cells were isolated by negative selection on AutoMACS (Miltenyi Biotec, San Diego, CA, USA) using an isolation kit from Miltenyi Biotec. T cells were activated using CD3/CD28 T-cell activator (25 μL/mL, Stemcell, Vancouver, BC, CA) and IL-2 (150 IU/mL, PeproTech, Bionity, Rocky Hill, CT, USA) in freshly prepared RPMI for 48–72 h prior to T cell culture for exosome production.

*Radioactive labeling and purification:* Exosomes isolated from supernatants of the cell lines listed in Table 1 or primary human T cell-derived exosomes were radioactively labeled with 0.5mCi ^125^I (Perkin Elmer, Waltman, MA, USA) using the chloramine-T method and purified on an Illustra NAP-5 (GE Healthcare, Piscataway, NJ, USA) column eluting with PBS. Bovine serum albumin (Sigma, St. Louis, MO, USA) was labeled with ^131^I using the chloramine-T method or ^99m^ Tc (GE Healthcare) using the stannous tartrate method. Both I-Alb and Tc-Alb were purified on a column of G-10 Sephadex (GE Healthcare).

*Mice:* 8-week-old CD-1 male mice (Charles River) were kept in a 12/12-h light/dark cycle and given ad lib water and food. All studies were performed under protocols approved by the Veterans Affairs Puget Sound Health Care System’s IACUC, a facility accredited by the Association for Assessment and Accreditation Laboratory Animal Care International (AAALAC).

*IV time curve:* Mice were anesthetized by giving an intraperitoneal (ip) injection of 0.15–0.2 mL 40% urethane. The left jugular vein was exposed and an injection of 0.2 mL lactated Ringer’s solution containing 1 × 10^6^ cpm of a radioactively labeled exosome and 1 × 10^6^ cpm of a radioactively labeled albumin was into the left jugular vein. At time points between 1 and 60 min, blood was collected from the carotid artery. Blood was centrifuged at 5400× *g* for 10 min and 50 uL serum collected. The brain, liver, lung, kidney and spleen were removed and weighed. All tissues and serum were placed into a gamma counter and the levels of radioactivity were measured. Results for serum were expressed as the percent of the injected dose per ml of blood (%Inj/mL). Results for tissues were expressed as the tissue/serum ratio in units of μL/g. For each individual tissue, its ratio for radioactive albumin were subtracted from its ratio for the radioactive exosome, yielding the “delta” value which reflected extravascular or tissue sequestration. Subtracting albumin removes the vascular and leakage contributions from the exosome tissue/serum ratios, yielding a better estimate of the selective sequestration of the exosome.

*Lipopolysaccharide (LPS) preparation and treatment:* LPS induces inflammation by activating the innate immune system. Mice were given an ip injection of 0.2 mL of 0.9% NaCl solution with half the injections containing 3 mg/kg LPS (Sigma, St. Louis, MO, USA). Mice received 3 such injections at t = 0, 6, 24 h. At 28 h after the first injection, mice were anesthetized with urethane and studied as outlined above, except time points after radioactive injection ranged from 1–20 min. Whole brain did not include the olfactory bulb.

*Effects of mannose-6-phosphate (M6P) and wheatgerm agglutinin (WGA) on tissue sequestration:* M6P blocks the uptake of substances that use the M6P receptor for sequestration. WGA enhances endocytosis and transcytosis of substances that bind to cell-surface glycoproteins containing N-acetylglucosamine or sialic acid. Mice were given an ip injection of 0.15–0.2 mL 40% urethane. The left jugular vein and the right carotid artery were exposed. An injection of 0.2 mL lactated Ringer’s solution containing 1 × 10^6^ cpm of the radioactive exosome and 1 × 10^6^ albumin was given into the jugular vein at t = 0. In some mice, this injection contained either 10 ug wheatgerm agglutinin (WGA; Sigma) or 80 ug mannose-6-phosphate (M6P; Sigma). At t = 20 min, carotid artery blood and tissues were collected, and the results expressed as described above.

*Statistical Analysis:* To calculate clearance from blood, the %Inj/mL values were transformed to log values and regressed against time. We compared linear and nonlinear (one phase decay) models using the Prism statistical package (GraphPad Inc, San Diego, CA, USA) and report the best fit. Mice were studied over a 60 min period. Blood-to-tissue unidirectional influx rates (Ki’s; units of μL/g-min) were calculated by regressing the delta values (tissue/serum ratios corrected for the albumin space) against exposure time with multiple-time regression analysis [32,33]. This method yields the Ki and also the initial volume of distribution in the tissue (Vi; units of μL/g). Each regression is reported with its n, r^2^, and *p* value. Means are reported with their standard error terms and n’s. Study period was 60 min. Means are reported with their standard errors and n/group.

Path analysis based on methods as outlined by Baron and Kenny [34] was used to further explore relations among exosome-tissue interactions. Such analysis produces a graph with pairs of tissues connected, with those pairs with the strongest statistical connection taken to be more closely related. No directionality or dependency among the tissues was assumed. To probe for relations among the tissues, the delta tissue/serum values from the controls of the WGA study for each tissue was regressed against each of the other tissues using Spearman correlation and the values for r^2^ ranked from high to low. Starting with the pairs with the highest r^2^ values, pairs were graphed unless such graphing would produce a closed loop. Pairs in the resulting graph with higher r^2^ values are taken to be more closely related than those with lower r^2^ values or that are connected indirectly through other tissues. To probe for relations among exosomes, delta tissue/serum values from the controls of the WGA study for each exosome was regressed against each of the other exosomes using Spearman correlations.

## 5. Conclusions

Exosomes were readily sequestered by liver, kidney, spleen, and lung, and sequestration by these tissues of various exosomes was modified by LPS and WGA. However, patterns of sequestration and responses to LPS and WGA varied greatly among the exosomes. No evidence for a universal binding site controlling exosome-tissue interactions was obtained. Both tissues and exosomes differed in sequestration patterns, but they clustered reflecting similar binding and sequestration patterns. The exosome-tissue binding sites are likely to be highly similar between brain and lung and between spleen and liver. Likewise, exosomes not related by source of origin (i.e., mouse vs. human; cancer vs. non-cancer) likely cluster by using a common set of binding sites for tissue interactions.

## Figures and Tables

**Table 1:** Exosome Sources and Producer Cell Lines Characteristics *.

Designation	Species	Non-Cancerous/Cancer	Tissue
J774A.1	Mouse	Non-Cancerous	Macrophage
NIH-3T3	Mouse	Non-Cancerous	Fibroblast
SCCVII	Mouse	Cancer	Oral Squamous
Primary T Cell	Human	Non-Cancerous	T Cell
HaCaT	Human	Non-Cancerous	Keratinocyte
MEL526	Human	Cancer	Melanoma
MDA-MB-231	Human	Cancer	Breast
PCI-30	Human	Cancer	Head & Neck
SCC-90	Human	Cancer	Head & Neck
Kasumi	Human	Cancer	Leukemia

* Exosomes were isolated as described in Methods by SEC from the supernatants of the above listed cell lines.

**Table 2:** Means (x), standard deviations (SD) and number per cell (n) for each exosome and tissue. Results are from control mice used in the WGA experiment.

	Brain			Liver			Lung			Kidney			Spleen		
	X	SD	n	X	SD	n	X	SD	n	X	SD	n	X	SD	X
J774.A1	5.82	0.47	10	537	16.9	10	121	9.8	10	75.3	6.3	10	599	39.6	10
NIH-3T3	2.35	0.55	9	507	92.5	9	21.6	13.9	9	-34	10.3	9	532	76.9	9
Primary T Cell	12.1	1.6	8	2157	255	8	438	105	8	466	123	8	1018	208	8
HaCaT	1.79	0.33	10	282	14.2	10	13.9	5.7	10	-28.3	8.8	10	302	30.1	10
SCCVII	5.38	0.37	8	718	133	8	93.8	13.1	8	-48.8	14.8	8	1097	71.5	8
MEL526	5.18	2.32	8	419	62	8	76.5	38.8	8	55.8	18.5	8	514	44.2	8
MDA-MB-231	8.6	3.7	9	452	50	9	119	51.5	9	79.9	33.8	9	402	64	9
PCI-30	8.41	0.77	10	674	72.1	10	114.9	9.7	10	62.6	10	10	829	90.9	10
SCC-90	6.54	0.47	10	981	50.2	10	99.1	0.3	10	104	9	10	751	73	10
Kasumi	4.82	0.21	8	518	63	8	72.2	36.7	8	73.8	24.3	8	678	70.8	8

## Data Availability

All data generated or analyzed are included in this article. Raw data is available upon reasonable request.

## Supplementary Materials

The following supporting information can be downloaded at: https://www.mdpi.com/article/10.3390/ijms232012513/s1. Reference [35] is cited in the supplementary materials.
