# Supplementary material for: Characteristics of Exosomes and the Vascular Landscape Regulate Exosome Sequestration by Peripheral Tissues and Brain
Source: Int J Mol Sci. 2022 Oct 19;23(20):12513. doi: 10.3390/ijms232012513 (PMC9603979; doi:10.3390/ijms232012513)
Supplement: Supplementary file 1 [file ijms-23-12513-s001.zip › ijms-1854621-supplementary.pdf]

Supplemental Figures and Legends

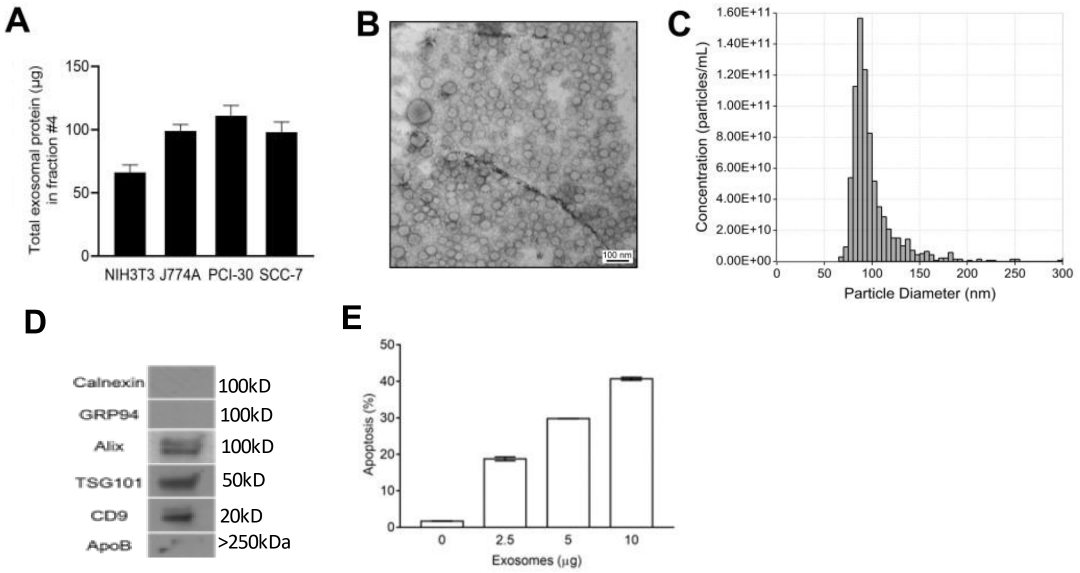

Figure S1. Methods used for the characterization of exosomes isolated by SEC (in fraction #4) from the supernatants of the 10 cell lines. Shown are exosomes isolated from the supernatant of PCI-13 cells. **A**. Examples of exosome recovery in Fr#4 for four different cell lines shown as total exosome protein (TEP). **B**. Transmission electron microscopy (TEM): the scale bar = 100nm. **C**. qNano to measure vesicle size and concentration. **D**. Western blots to show the absence of cytosolic proteins (calnexin, GRP94) the presence of endocytic proteins (TSG101, ALIX), and CD9 tetraspanin. **E**. Apoptosis induced in CD8+ T cells by PCI 13 exosomes (Annexin binding assay). Panels B, C and E were originally published in [35].

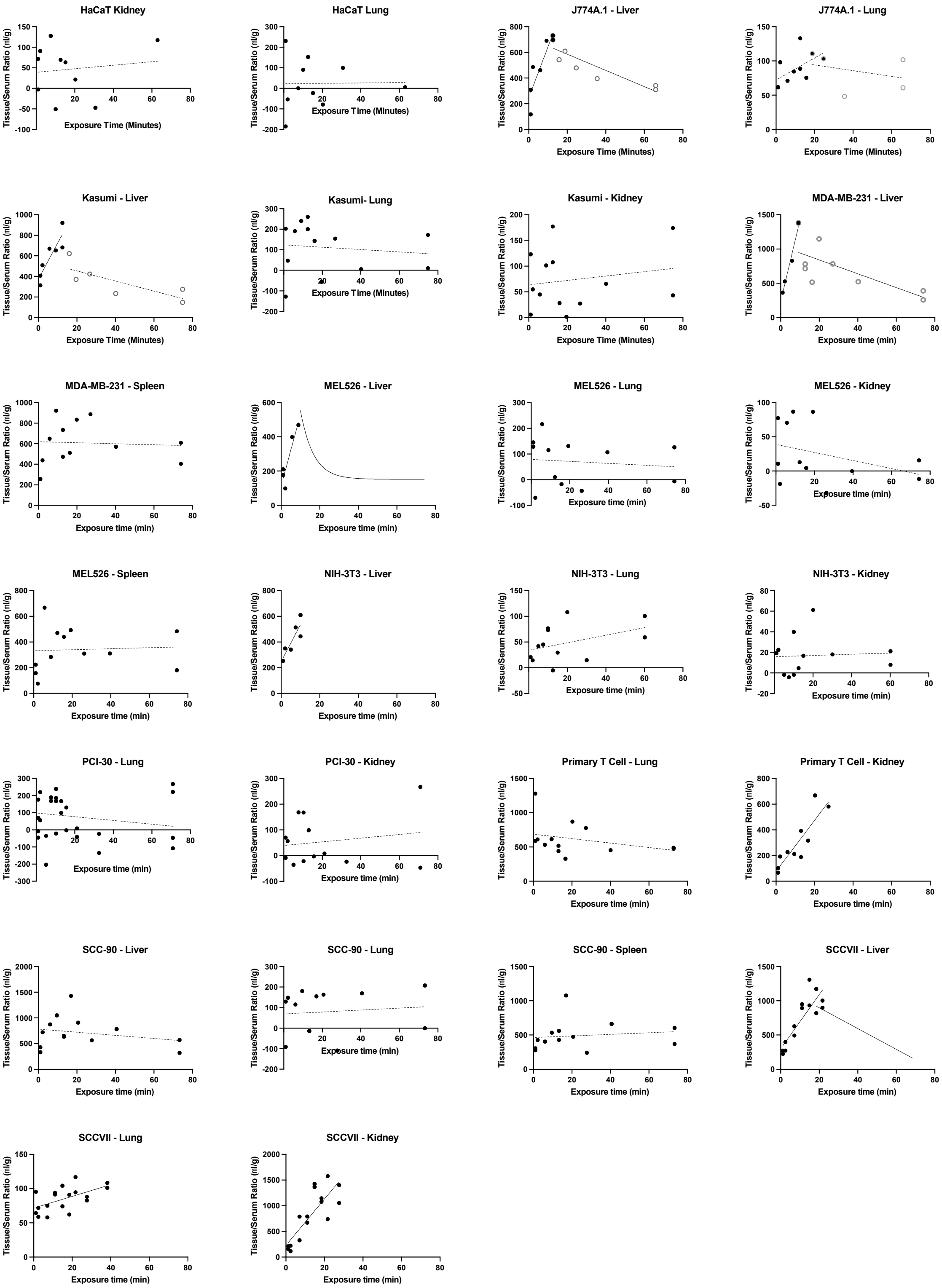
HaCaT Kidney
HaCaT Lung
J774A.1 - Liver
J774A.1 - Lung
Kasumi - Liver
Kasumi- Lung
Kasumi - Kidney
MDA-MB-231 - Liver
MDA-MB-231 - Spleen
MEL526 - Liver
MEL526 - Lung
MEL526 - Kidney
MEL526 - Spleen
NIH-3T3 - Liver
NIH-3T3 - Lung
NIH-3T3 - Kidney
PCI-30 - Lung
PCI-30 - Kidney
Primary T Cell - Lung
Primary T Cell - Kidney
SCC-90 - Liver
SCC-90 - Lung
SCC-90 - Spleen
SCCVII - Liver
SCCVII - Lung
SCCVII - Kidney
Tissue/Serum Ratio (ml/g)
Exposure Time (Minutes)
Exposure time (min)

Figure S2. Regressions analysis for exosome-tissues pairs not illustrated in figures 1-4. Solid lines are statistically significant correlations between tissue/serum ratios and exposure times and broken lines are not statistically significant.
